# Relationship between synthesis method–crystal structure–melting properties in co­crystals: the case of caffeine–citric acid

**DOI:** 10.1107/S205322962400319X

**Published:** 2024-05-07

**Authors:** Mathieu Guerain, Hubert Chevreau, Yannick Guinet, Laurent Paccou, Erik Elkaïm, Alain Hédoux

**Affiliations:** a Université Lille, CNRS, INRA, ENSCL, UMR 8207 – UMET – Unité Matériaux et Transformations, F-59650 Villeneuve d’Ascq, France; b Synchrotron SOLEIL, L’Orme des Merisiers, Saint-Aubin, BP 48, 91192 Gif-sur-Yvette, France; University of Aveiro, Portugal

**Keywords:** GALLOP, powder X-ray diffraction, caffeine, citric acid, cocrystal, DFT, synchrotron, polymorph, synthesis

## Abstract

The influence of the crystal synthesis method on the crystallographic structure of caffeine–citric acid co­crystals was analyzed thanks to the synthesis of a new polymorphic form of the cocrystal. In order to com­pare the new form to the already known forms, the crystal structure of the new cocrystal (C_8_H_10_N_4_O_2_·C_6_H_8_O_7_) was solved by powder X-ray diffraction thanks to synchrotron experiments. The structure determination was performed using ‘GALLOP’, a recently developed hybrid approach based on a local optimization with a particle swarm optimizer, particularly powerful when applied to the structure resolution of materials of pharmaceutical inter­est, com­pared to classical Monte-Carlo simulated annealing. The final structure was obtained through Rietveld refinement, and first-principles density functional theory (DFT) calculations were used to locate the H atoms. The symmetry is triclinic with the space group *P*  and contains one mol­ecule of caffeine and one mol­ecule of citric acid per asymmetric unit. The crystallographic structure of this cocrystal involves different hydrogen-bond associations com­pared to the already known structures. The analysis of these hydrogen bonds indicates that the cocrystal obtained here is less stable than the co­crystals already identified in the literature. This analysis is confirmed by the determination of the melting point of this cocrystal, which is lower than that of the previously known co­crystals.

## Abstract 2

A new polymorphic form of the caffeine–citric acid cocrystal, solved by powder X-ray diffraction thanks to synchrotron experiments, was com­pared with the already known forms. An analysis of the hydrogen bonding indicates that the cocrystal obtained here is less stable than the co­crystals already identified in the literature.

## Introduction

In recent years, the design of functional pharmaceutical mol­ecular materials by the cocrystallization technique has attracted increasing inter­est (Friščić & Jones, 2010 ▸) when other classical approaches based, for example, on salt formation or metastable polymorphs are not possible. The significant growth of this approach stems from the fact that numerous newly synthesized active pharmaceutical ingredients (APIs) in crystalline form exhibit insufficient solubility and bioavailability, constraining their therapeutic efficacy. Choosing a highly water-soluble coformer to construct an assembly of multiple neutral chemical species in the same crystal structure *via* weak supra­molecular inter­actions of various nature, such as van der Waals, hydrogen, halogen or π–π bonds, makes it possible to improve the bioavailability of APIs while preserving the physical stability intrinsic to the crystalline state. Pharmaceutical co­crystals generally consist of an API and a coformer present in the same crystal structure (Friščić & Jones, 2010 ▸; Vishweshwar *et al.*, 2006 ▸; Schultheiss & Newman, 2009 ▸; Brittain, 2013 ▸; Childs *et al.*, 2009 ▸), for example, paracetamol–piperazine (Oswald & Pulham, 2008 ▸), ibu­pro­fen–nicotinamide (Berry *et al.*, 2008 ▸; Guerain, Guinet *et al.*, 2020 ▸), carbamazepine–saccharin (Fleischman *et al.*, 2003 ▸), carbamazepine–dl-tartaric acid (Guerain, Derollez *et al.*, 2020 ▸), *etc*. These multicom­ponent materials in the crystalline solid state have an obvious inter­est in terms of stability, but also to improve many physicochemical properties of an API, such as aqueous solubility, dissolution, hygroscopicity or bioavailability. However, the discovery and preparation of new co­crystals remains empirical and is still based on trial and error (ter Horst *et al.*, 2009 ▸). Cocrystallization can be achieved by many different techniques, such as crystallization in solution, milling, milling assisted by a solvent, use of supercritical fluids or sonocrystallization, which may lead to different crystalline polymorphs in an uncontrolled manner (Schultheiss & Newman, 2009 ▸; ter Horst *et al.*, 2009 ▸; Karimi-Jafari *et al.*, 2018 ▸). It is worth noting that the preparation method has a direct influence on the structure of the cocrystal, which itself has a direct influence on the properties (Guerain, Derollez *et al.*, 2020 ▸; Guerain, Guinet *et al.*, 2020 ▸; Karki *et al.*, 2007 ▸; Smit & Hagen, 2015 ▸; Fleischman *et al.*, 2003 ▸). 
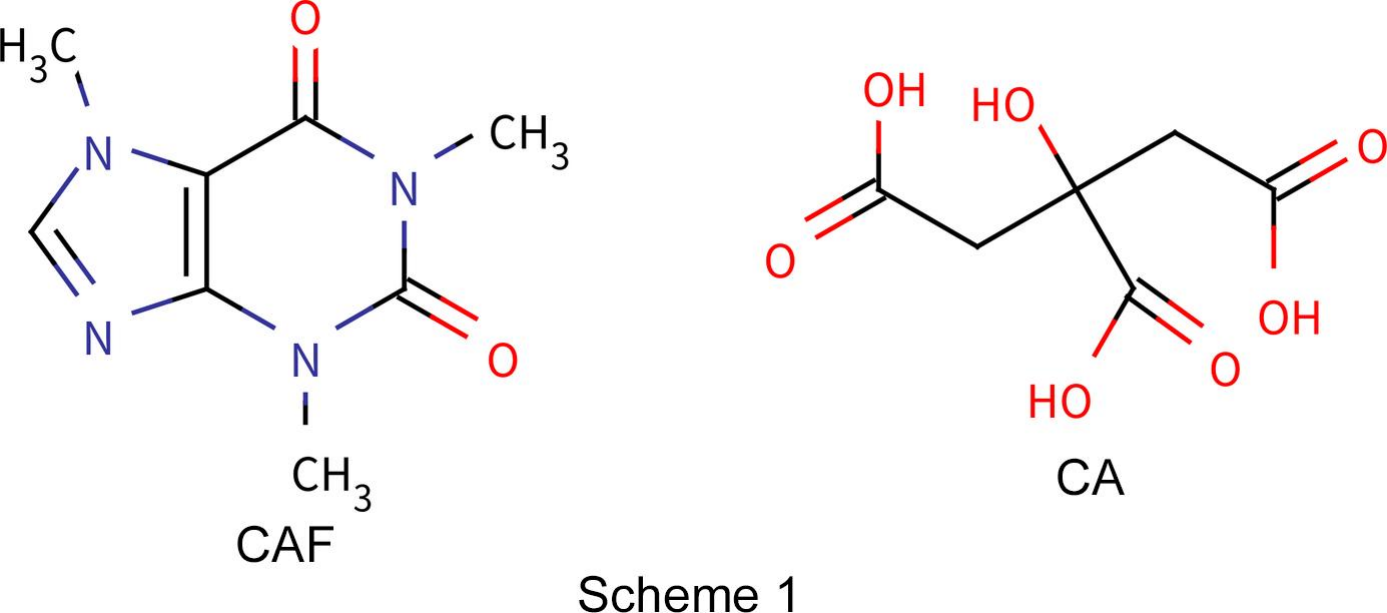


**Figure 1:**
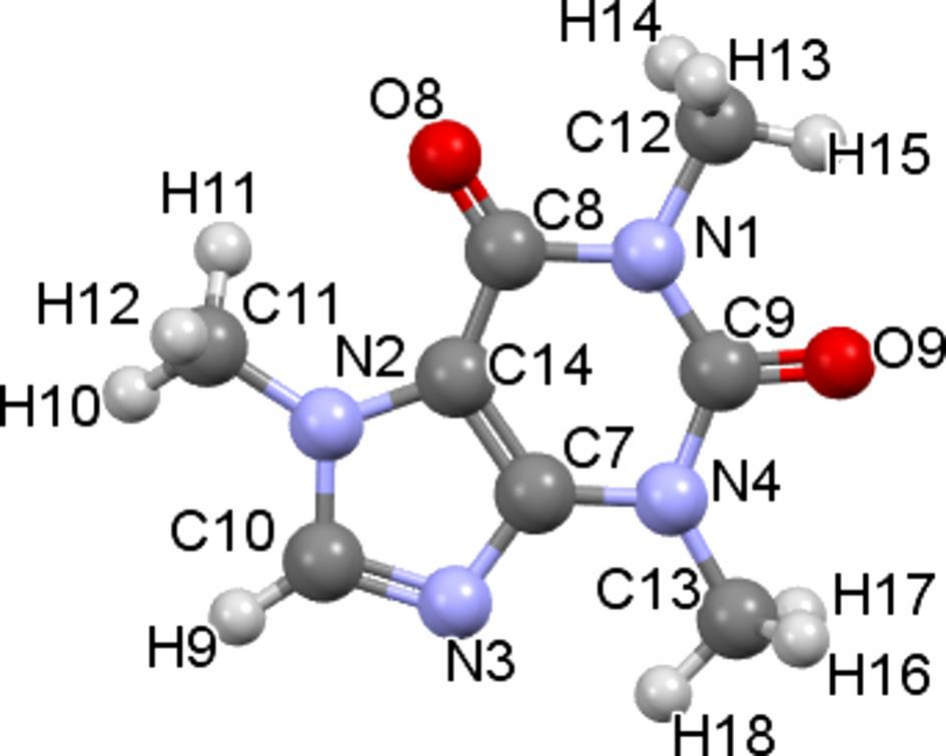
The mol­ecular structure of caffeine. C atoms are shown in black, N atoms in blue, O atoms in red and H atoms in white.

**Figure 2:**
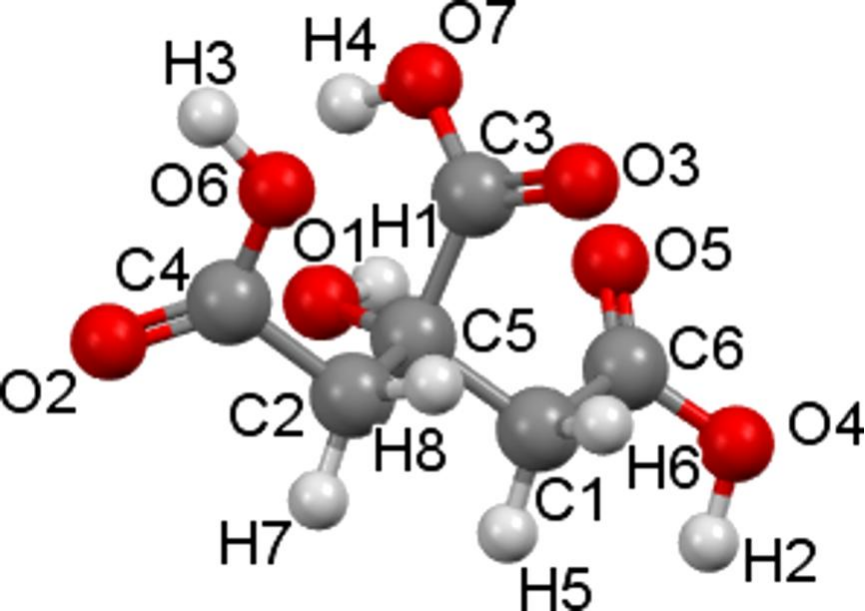
The mol­ecular structure of citric acid. The atomic colour codes are the same as in Fig. 1 ▸.

Caffeine (CAF, C_8_H_10_N_4_O_2_, 1,3,7-trimethyl-2,3,6,7-tetra­hydro-1*H*-purine-2,6-dione, see Fig. 1 ▸) is a xanthine alkaloid psychoactive stimulant drug and has been reported to crystallize as two polymorphic forms (Enright *et al.*, 2007 ▸) and one hemihydrate (Edwards *et al.*, 1997 ▸). Citric acid (CA, C_6_H_8_O_7_, 2-hy­droxy­propane-1,2,3-tri­carb­oxy­lic acid, see Fig. 2 ▸) is a crystalline organic acid which is often used as a coformer for cocrystallization. These two mol­ecules form two known co­crystals.

(i) The first polymorph was synthesized by milling together caffeine and citric acid with a 1:1 molar ratio (Karki *et al.*, 2007 ▸). It is reported under the reference code KIGKER in the Cambridge Structural Database (CSD; Groom *et al.*, 2016 ▸). The authors mention that milling the mixture with or without liquid leads to the same triclinic structure (space group No. 2, *P* 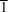 ) with the following unit-cell parameters: *a* = 7.38740 (10), *b* = 8.3967 (2), *c* = 13.5053 (3) Å, α = 91.3330 (10), β = 99.0400 (10), γ = 99.5880 (10)° and *V* = 814.716 Å^3^.

(ii) The second polymorph (CSD refcode KIGKER01) was synthesized by slow evaporation from a saturated solution of caffeine and citric acid (at about 30 °C) in a 1:1 molar ratio in chloro­form/methanol (1:1 *v*/*v*) (Smit & Hagen, 2015 ▸). The crystalline symmetry of the resulting cocrystal is monoclinic (space group No. 14, *P*2_1_/*c*) with the following unit-cell parameters: *a* = 13.7783 (8), *b* = 12.3149 (8), *c* = 9.6587 (6) Å, β = 92.854 (4)° and *V* = 1636.84 Å^3^. The melting tem­per­a­ture of this cocrystal is 158.9 °C.

In this work, CAF–CA co­crystals have been synthesized from caffeine monohydrate and citric acid from milling and solvent evaporation (Scheme 1). The present article aims to highlight the influence of the synthesis method on the hydrogen-bond association, crystallographic structure and structural disorder of CAF–CA co­crystals, and the consequences on their melting properties. For this, it was necessary to solve the crystallographic structure of a new CAF–CA cocrystal obtained by solvent evaporation to com­pare it with the already known co­crystals (Berry *et al.*, 2008 ▸; Lemmerer *et al.*, 2013 ▸; Surov *et al.*, 2023 ▸; Guerain, Guinet *et al.*, 2020 ▸). The structure of the new cocrystal was solved *ab initio* from powder X-ray diffraction using a recently developed hybrid algorithm, namely, GALLOP, based on a local optimization with a particle swarm optimizer (Spillman & Shankland, 2021 ▸). This approach was com­pared to classical Monte-Carlo simulated annealing algorithms based on global optimization, and the structure was confirmed by Rietveld refinement. H atoms were located by first-principles density functional theory (DFT) calculations.

## Experimental

### Materials

Caffeine monohydrate (purity higher than 98.5%) was purchased from ACROS and the material was used without any purification. An analysis of the powder X-ray diffraction pattern showed that the commercial material is in the stable monoclinic phase (CSD refcode CAFINE01; Edwards *et al.*, 1997 ▸).

Citric Acid (purity higher than 99.5%) was purchased from Sigma–Aldrich and the material was used without any purification. An analysis of the powder X-ray diffraction pattern showed that the commercial material is in the stable ortho­rhom­bic phase (CSD refcode CITARC01; King *et al.*, 2011 ▸).

### Cocrystal synthesis

Cocrystals were synthesized using two different methods, *i.e.* by milling and by evaporation from a solution.

The milling was performed with a 1:1 molar mixture cor­responding to 212 mg of caffeine monohydrate and 192 mg of citric acid on a vibrating-mill Retsch MM400 at 30 Hz. ZrO_2_ milling jars of 10 cm^3^ were used, with one ball (diameter 10 mm). The milling time was set at 30 min. We took care to alternate milling periods (typically 10 min) with pause periods (typically 5 min) in order to limit the mechanical heating of the sample. No liquid was used for assistance.

Cocrystals were also synthesized by slow evaporation (at about 30°C) from a 1:1 molar stoichiometric mixture of 212 mg of caffeine monohydrate and 192 mg of citric acid dissolved in an aceto­nitrile–ethanol mixture (1:1 *v*/*v*).

### Raman spectroscopy analysis

Raman spectroscopy investigations were performed using two spectrometers, depending on the investigated spectral domain.

Low-wavenumber Raman spectra were collected in the 5–300 cm^−1^ range using a highly dispersive XY Dilor Raman spectrometer to analyse the non-polarized back-scattered light. The spectrometer is com­posed of a triple monochromator in a configuration characterized by a focal length of 800 mm. The choice of experimental conditions (incident radiation from a mixed argon–krypton coherent laser selected at 514.5 nm, and entrance and exit slit widths opened at 150 µm) allows the rejection of the elastic scattering below 5 cm^−1^ without additional filters, and gives a spectral resolution of about 1 cm^−1^ in the 5–300 cm^−1^ region. This spectrometer was only used for analyzing the low-frequency region characterized by a relatively intense Raman signal, spectra being taken in 120 s. This spectral region gives the opportunity to analyse lattice modes, giving the crystalline fingerprint of polymorphic forms.

High-frequency (2700–3200 cm^−1^) Raman spectra were col­lected using an InVia Renishaw micro-Raman spectrometer. The laser line (514.5 nm line from a Fandango Cobolt laser) was focused on the powder sample *via* a Leica X50 objective providing the signal within a volume of about 150 µm^3^. The sample tem­per­a­ture was controlled by placing the sample in a THMS 600 Linkam tem­per­a­ture device.

### Synchrotron experiments and data collection

The powder X-ray diffraction (PXRD) patterns were measured at the high-resolution powder diffraction beamline CRISTAL at the Synchrotron SOLEIL in France. The beamline is equipped with a 1D MYTHEN2 X detector. The selected energy was 17 keV, corresponding to a wavelength (λ) of 0.7289 Å and a NIST LaB_6_ 660a sample was used for calibration. The cocrystal powder was enclosed in a glass capillary (diameter 0.5 mm) and mounted on the goniometer head. The capillary was rotated during the experiments to reduce the effect of a possible preferential orientation. In order to limit radiation damage, data were collected at room tem­per­a­ture in the 2.5–50° 2θ range in less than 2 min.

### Density functional theory (DFT) calculations

First-principles calculations were performed using the pro­gram *pw.x*, as implemented in the package *Quantum ESPRESSO* (Giannozzi *et al.*, 2009 ▸, 2017 ▸). The generalized gradient approximation (GGA) with the Perdew–Burke–Ernzerhoff for solids (PBEsol) exchange correlation function was employed (Perdew *et al.*, 1996 ▸). Projector-augmented wave pseudopotentials for all elements (C, N, O and H) from the ‘precision’ Standard Solid State Pseudo-potential (SSSP) library were used in the calculations (Prandini *et al.*, 2018 ▸). The wave function cut-off energy was set to 60 Ryd and the supercell was sampled with a 2 × 3 × 4 Monkhorst–Pack k-point grid (Monkhorst & Pack, 1976 ▸). In order to calculate more accurately the van der Waals inter­actions, an empirical dispersion correction was included in the DFT calculations with the Grimme’s DFT-D3 scheme (Grimme *et al.*, 2010 ▸).

## Results

### Cocrystal synthesis and identification

**Figure 3:**
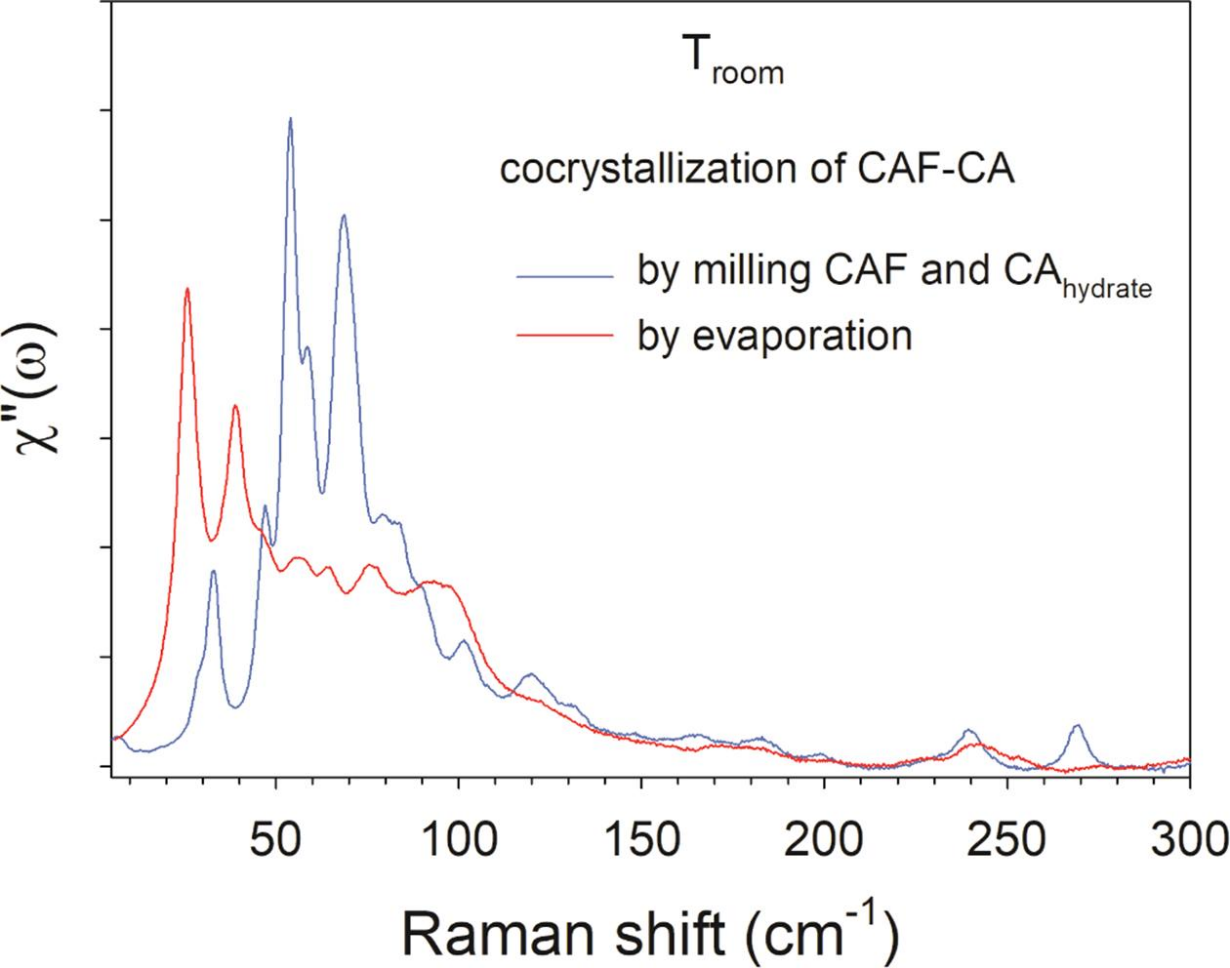
Raman susceptibility spectra of CAF–CA co­crystals prepared by milling (in blue) and by evaporation (in red).

The low-frequency Raman spectra (LFRS) in the 5–300 cm^−1^ region provide the crystalline fingerprints of the co­crystals. The difference in the structural description is clearly observed in the spectra of the lattice modes of the two co­crystals prepared by milling and solvent evaporation, plotted in Fig. 3 ▸, directly representative of their crystalline identity. The Raman spectrum collected for the co­crystals prepared by milling provides information in agreement with that published by Karki *et al.* (2007 ▸). By contrast, the spectrum of the co­crystals synthetized from solvent evaporation shows significant differences com­pared to the spectrum of the co­crystals prepared by milling and whose structure is already known.

An important consequence arising from these investigations is the evidence of a new crystalline form for caffeine–citric acid co­crystals, denoted CAF–CA [the previous known forms are called KIGKER and KIGKER01 with reference to the Cambridge Structural Database (CSD) refcodes]. Indeed, it turns out that this CAF–CA cocrystal does not correspond to that published by Karki *et al.* (2007 ▸) with CSD refcode KIGKER or to that published by Smit & Hagen (2015 ▸) with CSD refcode KIGKER01.

It was also impossible to reproduce the KIGKER01 cocrystal in our laboratory. Such results are not surprising because such cases have already been reported in the literature as ‘disappearing polymorphs’ (Hasa *et al.*, 2020 ▸; Dunitz & Bernstein, 1995 ▸; Blagden *et al.*, 1998 ▸). This is precisely the case with this system (caffeine and citric acid), in particular, which has been the subject of a dedicated publication (Hasa *et al.*, 2020 ▸). The authors mention several parameters which could lead to the ‘disappearance’ of caffeine–citric acid cocrystal polymorphs.

These parameters are difficult to control, and to report in the literature, during synthesis by both milling and solvent evaporation, and include atmospheric moisture in the laboratory and the possible existence of ‘invisible seeds’ which could ‘infect’ the laboratory and drive the crystallization toward a given polymorph (Hasa *et al.*, 2020 ▸; Dunitz & Bernstein, 1995 ▸; Blagden *et al.*, 1998 ▸). In our case, it is also possible that the evaporation rate of the solvent during the synthesis of the cocrystal is a parameter to consider, but this is also difficult to qu­antify and control.

In any case, to the best of our knowledge, the CAF–CA cocrystal is not referred to in the literature or in the following databases: CSD (Groom *et al.*, 2016 ▸), crystallographic open database (COD) (Gražulis *et al.*, 2009 ▸) and the PDF-2 database of the Inter­national Center for Diffraction Data (ICDD) (Gates-Rector & Blanton, 2019 ▸). It was therefore necessary to solve this cocrystal in order to be able to com­pare its crystal structure with the structures of the published co­crystals.

### Structure solution and refinement of the new CAF–CA cocrystal

The indexation of the data obtained at the Synchrotron SOLEIL was performed using *DICVOL* (Boultif & Louër, 2004 ▸), as implemented in the *FullProf* suite. The best solution suggests a triclinic symmetry with lattice parameters of *a* = 14.79, *b* = 8.95, *c* = 7.02 Å, α = 106.36, β = 95.84, γ = 97.47° and *V* = 876.12 Å^3^. The calculated figures of merit (de Wolff *et al.*, 1968 ▸; Smith & Snyder, 1979 ▸) are M(20) = 10.9 and F(20) = 61.9. A space-group determination indicates *P* 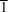 (No. 2), which has a higher frequency in the CSD, *i.e.* 25.2% *versus* 1% for *P*1 (No. 1) as of January 2023 (Groom *et al.*, 2016 ▸). Moreover, the KIGKER structure crystallizes in the space group *P* 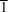 (No. 2) and exhibits a similar unit-cell volume of 814.716 Å^3^, the present structure exhibits a unit-cell volume of 848.639 (2) Å^3^. The KIGKER structure is built up from an asymmetric unit containing two CAF and two CA mol­ecules. The structure determination was thus performed using an asymmetric unit with this content.

The *ab initio* structure determination was performed with the recently developed hybrid algorithm GALLOP, which combines a local optimization with a particle swarm optimizer (Spillman & Shankland, 2021 ▸). Making use of graphics pro­cessing units (GPUs), this approach allows us to explore intelligently, through the particle swarm optimizer, several thousand starting positions of known mol­ecules followed by a local optimization. GALLOP requires Pawley fitting output files from *DASH*, *GSAS-II* or *Topas*, as well as the mol­ecule(s) described in the Z-matrix format produced by *DASH*. This makes it particularly suitable for solving the structures of new crystals of known mol­ecules. Here, the caffeine mol­ecule and the citric acid mol­ecule were retrieved from the CSD, *i.e.* from the monoclinic caffeine hydrate phase model (Sutor, 1958 ▸; Edwards *et al.*, 1997 ▸) and from the ortho­rhom­bic citric acid hydrate phase model (King *et al.*, 2011 ▸), respectively. The volume calculated from the indexation (*V* = 876.16 Å^3^) being similar to one of the co­crystals reported by Karki *et al.* (2007 ▸) (*V* = 814.7 Å^3^), one mol­ecule of caffeine and one mol­ecule of citric acid were introduced randomly in the unit cell. The calculation was performed with the GALLOP python API on google colab using a NVIDIA Tesla K80 GPU. Using the default parameters, *i.e.* a number of swarms of 10 and a number of particles per swarm of 10000 for the particle swarm optimizer, and a number of 500 iterations for the local optimization, the calculation lasts for approximately 6 min.

The so-obtained structure was com­pared to the two other structures obtained by well-established *SDPD* (Structure Determination from Powder Diffraction) programs based on rigid-bodies mol­ecules. Thus, the structure determination was also achieved with *DASH* (David *et al.*, 2006 ▸) and *FOX* (Favre-Nicolin & Černý, 2002 ▸). Contrary to GALLOP, which is based on local optimization, both programs are based on global-optimization algorithms using simulated annealing and parallel tempering algorithms, respectively, to solve the structure by performing trials in direct space. The best solutions obtained by these three programs are displayed in Fig. S1 in the supporting information, one can see they are in good agreement with one another. GALLOP is thus adapted to solve the structure of co­crystals from powder diffraction measured at the synchrotron. Moreover, the GALLOP calculation (without considering the set-up of the calculation) was performed in less than a minute, while a few hours were required for *DASH* and *FOX*. A thorough com­parison remains com­plicated as these codes are built up differently, indeed GALLOP runs on a GPU while *DASH* and *FOX* run on a CPU.

The positions of the H atoms were obtained by density functional theory (DFT) calculations. Using the structure determined from GALLOP, a ground-state calculation was performed allowing only the H atoms to move freely. The heavy atoms, *i.e.* carbon, nitro­gen and oxygen, were fixed in their positions, and the lattice parameters were also fixed.

**Figure 4:**
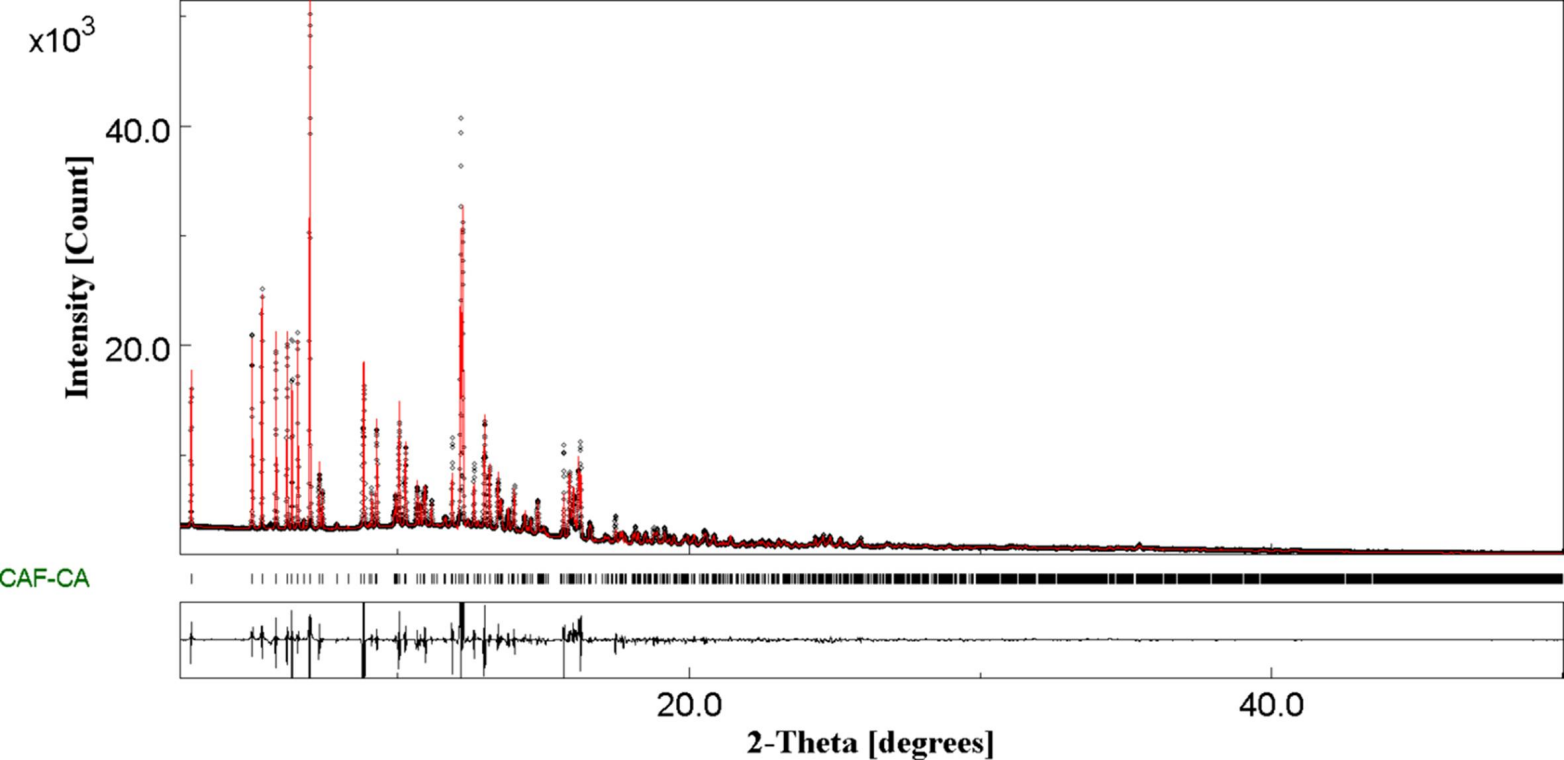
Final Rietveld plot of the CAF–CA co­crystals at room tem­per­a­ture between 2.5 and 50° (*MAUD* software; https://luttero.github.io/maud/). Observed intensities are indicated by dots, and solid lines represent the best-fit profile (upper trace) and the difference pattern (lower trace). The vertical bars correspond to the positions of the Bragg peaks.

Finally, a Rietveld refinement was performed to validate the model and to refine the structure with the experimental powder X-ray diffraction pattern (Fig. 4 ▸). The structure contains the position of the heavy atoms obtained from GALLOP and the H atoms obtained from DFT-D3 calculations. The Rietveld refinement was performed with the program *Jana2020* (Petrícek *et al.*, 2014 ▸) to generate the most accurate and com­plete CIF file possible. The lattice parameters and final conventional Rietveld factors after Rietveld refinement are available in Table 1 ▸, together with the crystallographic data, profile and structural parameters.

### Tem­per­a­ture dependence of the C—H stretching spectrum and melting properties of the co­crystals

**Figure 5:**
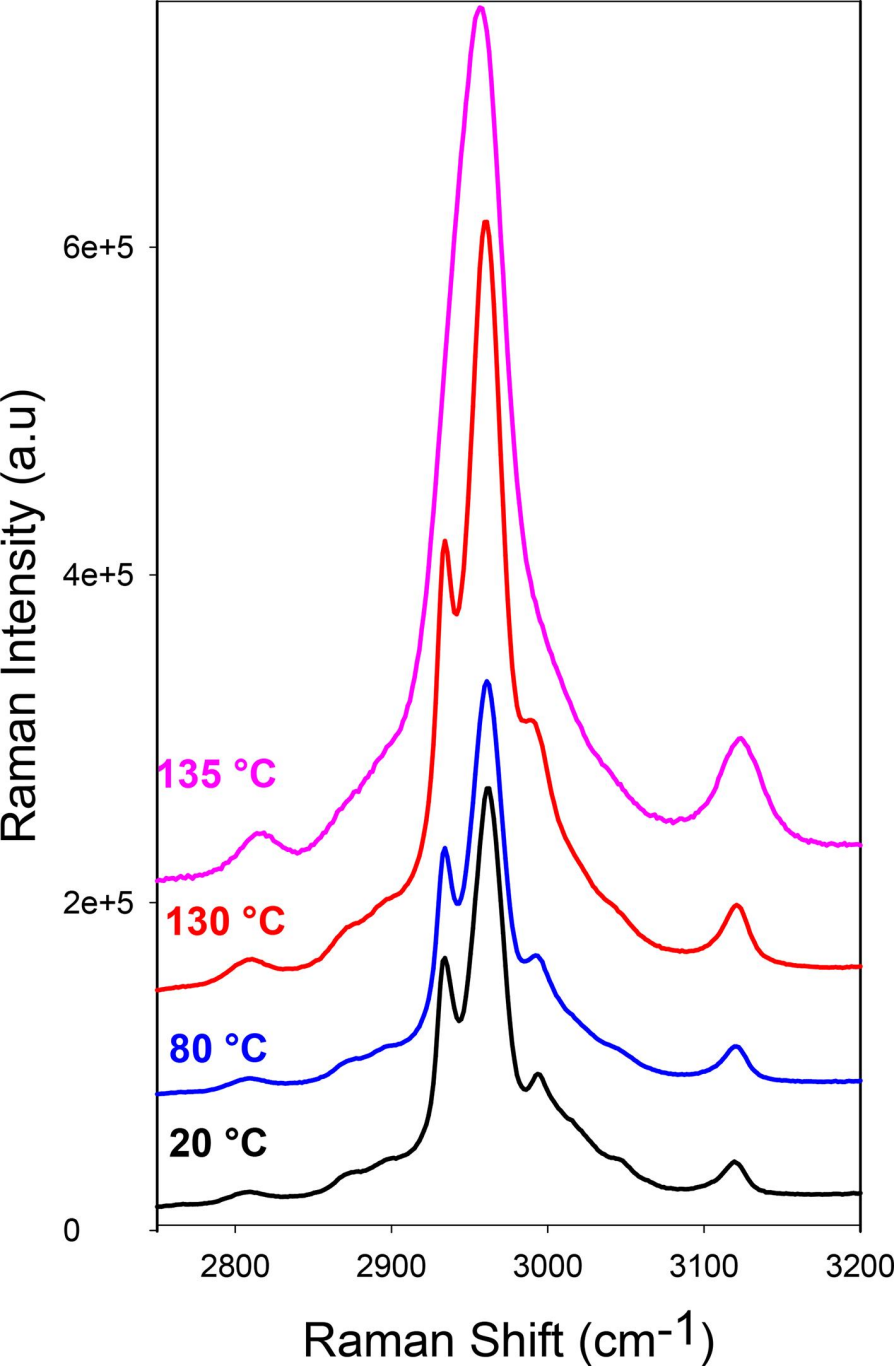
Tem­per­a­ture dependence of the Raman susceptibility spectra for CAF–CA co­crystals prepared by evaporation.

The melting tem­per­a­ture of the CAF–CA co­crystals was determined thanks to high-tem­per­a­ture Raman spectroscopy experiments. The Raman spectrum in the range 2700–3200 cm^−1^ corresponds to the spectrum of the C—H stretching vibrations. The tem­per­a­ture dependence of the spec­trum is plotted in Fig. 5 ▸. The C—H stretching region is dominated by a doublet clearly distinguishable between 20 and 130 °C. At 135 °C, the spectrum can be considered as the envelope of Raman bands observed at lower tem­per­a­ture, and the most intense Raman bands within the doublet have merged into a broadened band. It is well known that the C—H stretching region is almost tem­per­a­ture independent, except on either side of a phase transition (Hédoux, 2016 ▸; Hédoux *et al.*, 2011 ▸). Consequently, the very broad C—H stretching spectrum taken at 135 °C is typically mimicking the spectrum of the liquid, and reveals the melting of the cocrystalline form below 135 °C.

The melting tem­per­a­ture of KIGKER01 has been reported as 158.9°C (Smit & Hagen, 2015 ▸). The melting tem­per­a­ture of cocrystal KIGKER reported in the literature (Karki *et al.*, 2007 ▸) was also assessed in our work (see Fig. S2 in the supporting information) and is close to 161 °C. The melting point of the CAF–CA cocrystal is therefore 24 °C lower than the melting point of the KIGKER01 cocrystal and 26 °C lower than the melting point of the KIGKER cocrystal.

## Discussion

We com­pare here the structure of a new CAF–CA cocrystal synthesized by slow evaporation from aceto­nitrile/ethanol (denoted CAF–CA) with the co­crystals synthesized by ball milling (denoted KIGKER after its CSD refcode) and slow evaporation from chloro­form/methanol (denoted KIGKER01 after its CSD refcode).

The lattice parameters of CAF–CA, KIGKER and KIGKER01 are given in Table 2 ▸. Both CAF–CA and KIGKER are triclinic with *P* 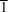 symmetry and a close unit-cell volume and *b* parameter, but different *a* and *c* parameters. The α angle is also very different between the two structures, since it is 15° higher for CAF–CA than for KIGKER. One can note that KIGKER01 has a very different crystallographic structure; it is monoclinic (*P*2_1_/*c*) with twice the unit-cell volume of the other structure.

**Figure 6:**
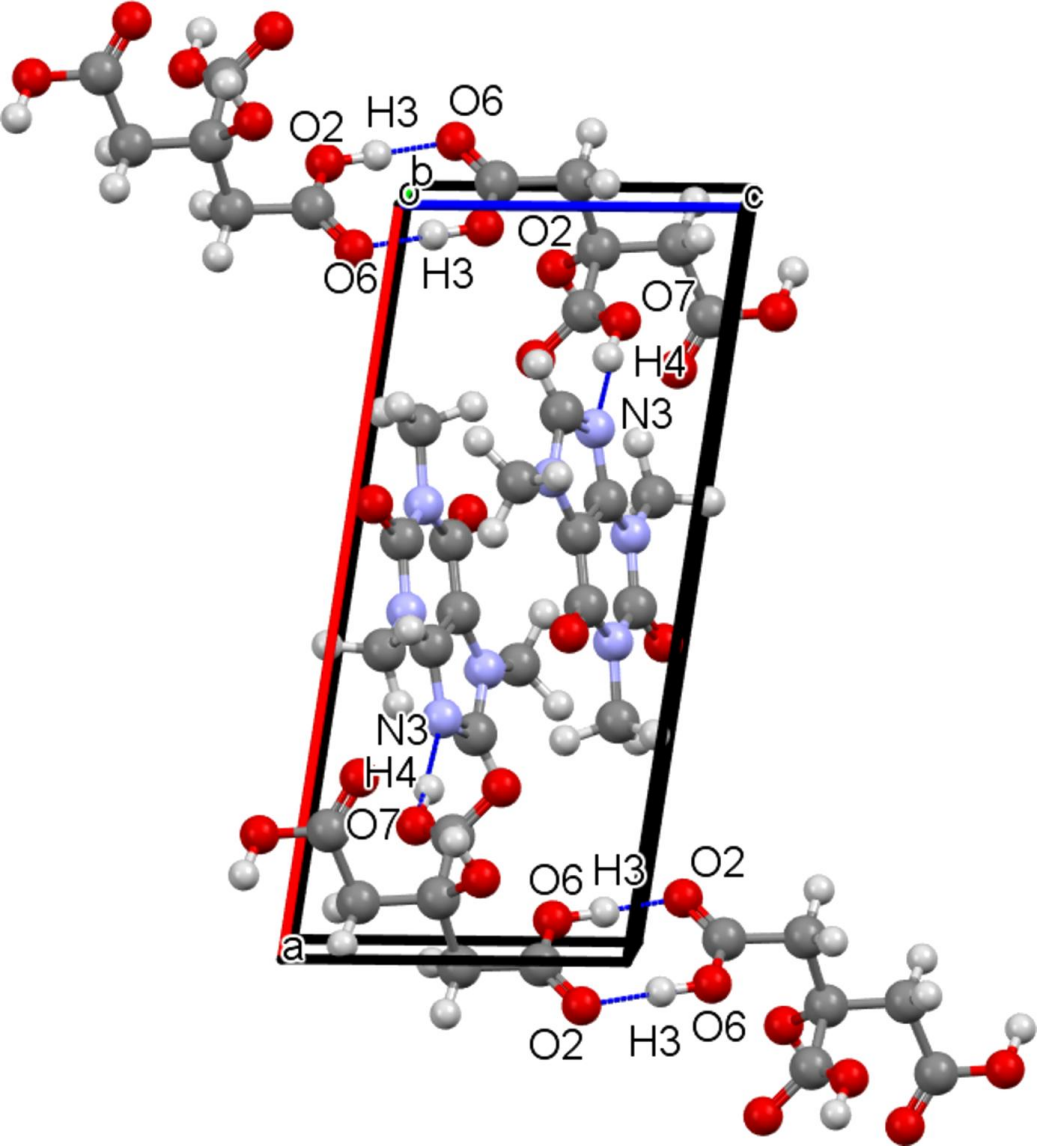
Visualization of the hydrogen-bond network of the CAF–CA cocrystal obtained by slow evaporation from aceto­nitrile–ethanol. Hydrogen bonds are represented by blue dotted lines.

**Figure 7:**
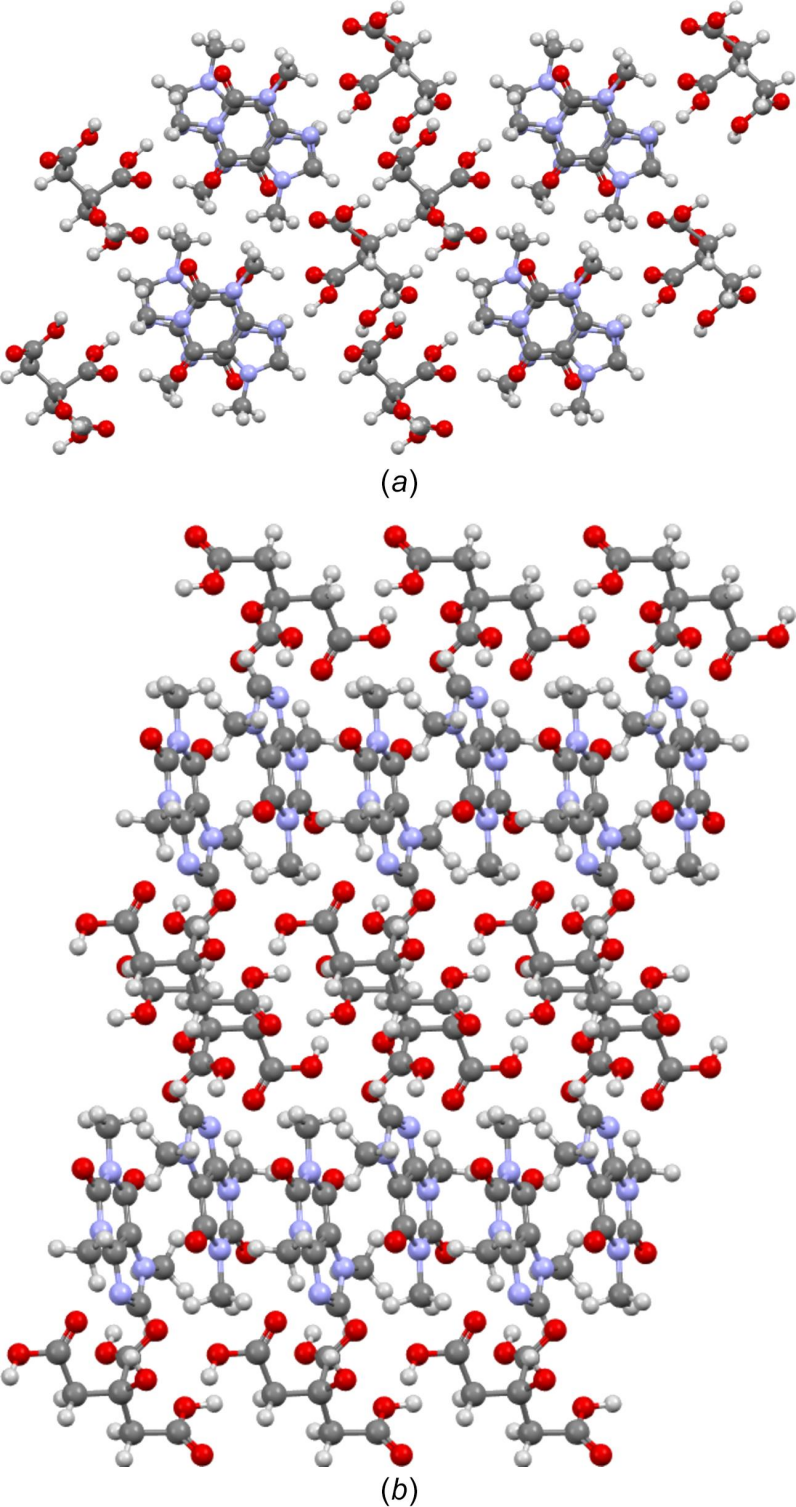
Projection of the unit cell of the CAF–CA cocrystal obtained by slow evaporation from aceto­nitrile–ethanol along (*a*) the [001] direction and (*b*) the [010] direction.

These differences are due to the structural arrangements of the mol­ecules. In the case of the CAF–CA cocrystal, one can see the formation of O—H⋯N (O7—H4⋯N3) hydrogen bonds between CA and CAF mol­ecules, but also an O—H⋯O (O6—H3⋯O2) hydrogen-bonded dimer binding the CA mol­ecules (see Fig. 6 ▸). Thus, the CA mol­ecules are stacked along the *c* axis through these hydrogen bonds. The CAF mol­ecules are also stacked along the *c* axis, with a 180° rotation between two mol­ecules. Along the *a* axis, there is an alternation between CAF and CA mol­ecules, which explains why the *a* parameter is the largest of the lattice parameters (Fig. 7 ▸).

**Figure 8:**
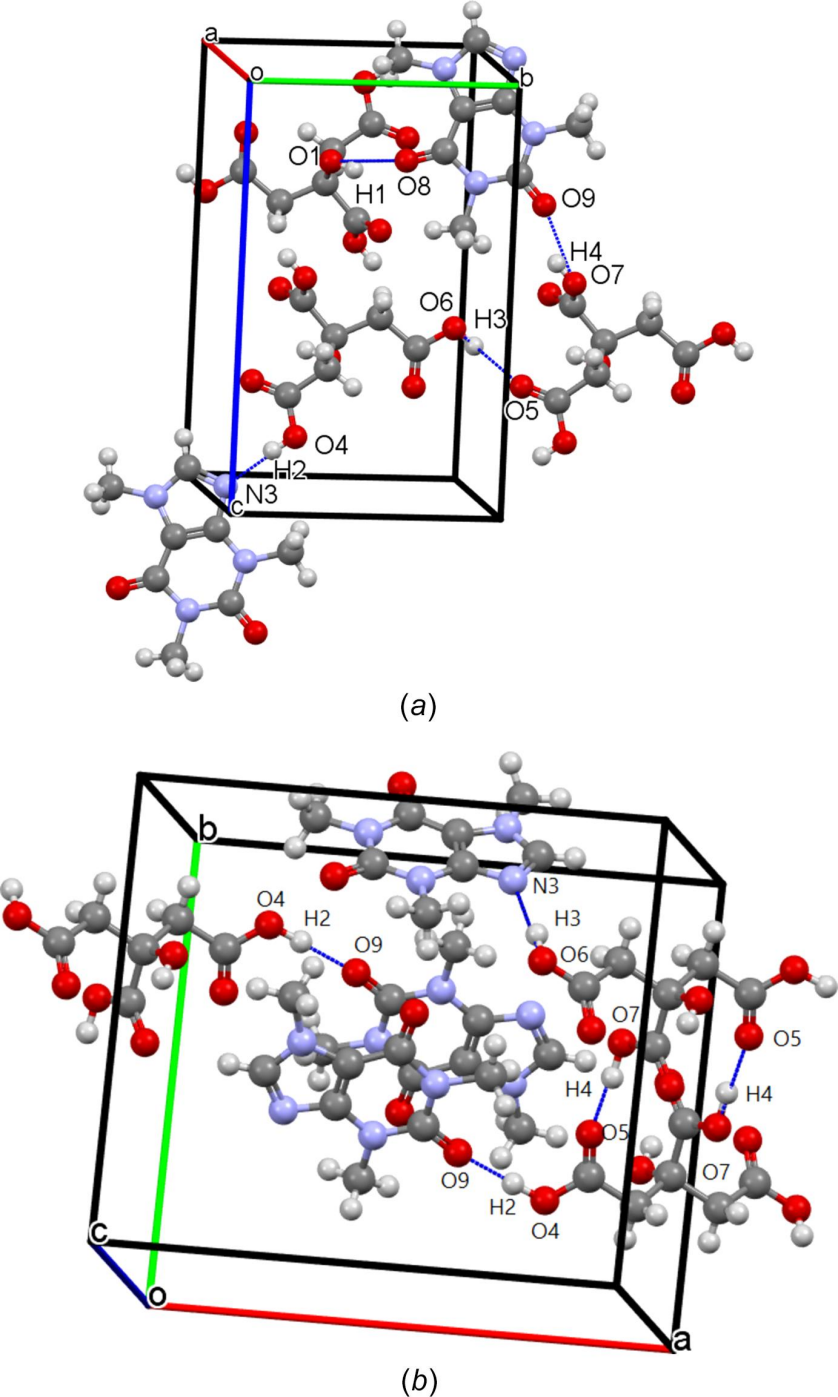
Visualization of the hydrogen-bond network for (*a*) the CAF–CA cocrystal KIGKER obtained by milling and (*b*) the CAF–CA cocrystal KIGKER01 obtained by slow evaporation from chloro­form–methanol. Hydrogen bonds are represented by blue dotted lines.

In co­crystals KIGKER and KIGKER01, no dimers are observed (see Fig. 8 ▸). For KIGKER, one can see O—H⋯O and O—H⋯N hydrogen bonds, namely, O6—H3⋯O5 hydrogen bonds between CA mol­ecules and O1—H1⋯O8, O7—H4⋯O9 and O4—H2⋯N3 hydrogen bonds between CA and CAF mol­ecules. For KIGKER01, O—H⋯O and O—H⋯N hydrogen bonds are also observed, namely, O7—H4⋯O5 hydrogen bonds bind the CA mol­ecules together, while O4—H2⋯O9 and O6—H3⋯N3 hydrogen bonds bind the CA and CAF mol­ecules.

**Figure 9:**
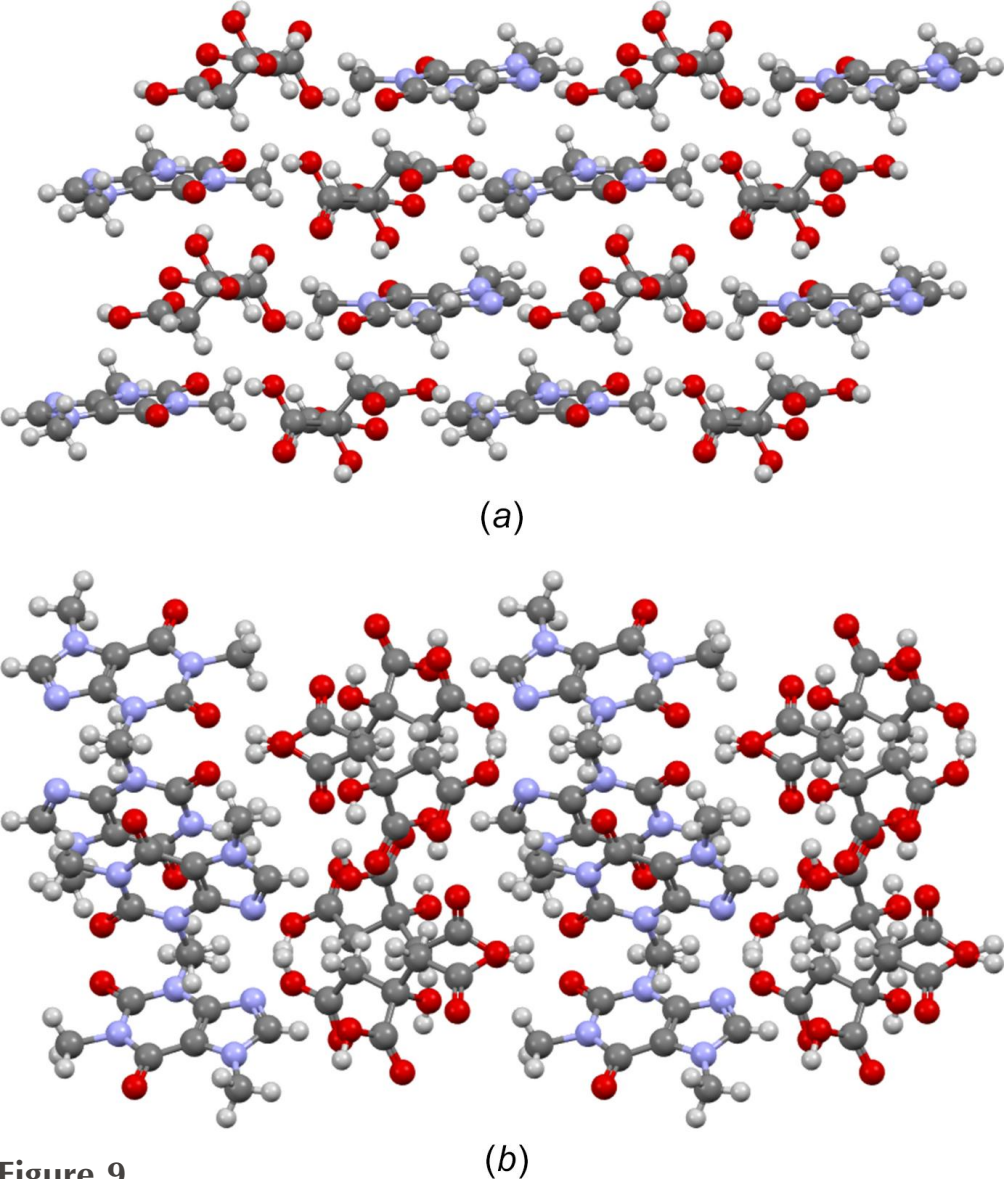
(*a*) Projection of the unit cell of cocrystal KIGKER along the [010] direction and (*b*) projection of cocrystal KIGKER01 along the [001] direction.

These different hydrogen-bond networks lead to different crystallographic structures of the CAF–CA co­crystals (see Fig. 9 ▸). For KIGKER, CAF and CA mol­ecules are stacked along the *b* axis, leading to a lattice parameter smaller than 10 Å in this direction. Along the *c* and *a* axes, there is an alternation between CAF and CA mol­ecules. This is an important difference with respect to the CAF–CA cocrystal, where the alternation exists only along one direction. The KIGKER01 structure is very different from that of the CAF–CA cocrystal with an alternation of CAF and CA mol­ecules along the *a* axis, but in particular a zigzag arrangement of CAF mol­ecules in the *bc* plane. This suggests that the chosen synthesis method, especially the tools utilized in preparing the com­pound, significantly shapes the resulting crystallographic structures and the hydrogen-bond network and, consequently, influences the physico-chemical properties.

It is well known that the bonding network influences the melting properties of an organic com­pound. Indeed, the melting point of the CAF–CA cocrystal (135 °C) is lower than those of KIGKER (161 °C) and KIGKER01 (158.9 °C), indicating that the CAF–CA crystal structure is less stable with regard to tem­per­a­ture. This was confirmed by ground-state DFT calculations (see Table 3 ▸). Such calculations were performed on the three co­crystals: CAF–CA, KIGKER and KIGKER01. The obtained energies are −1910962.41, −1911085.31 and −1911085.05 kJ mol^−1^, respectively. These calculations indicate the order of stability to be KIGKER > KIGKER01 >> CAF–CA, which is in good agreement with the melting tem­per­a­ture of the co­crystals. Further calculations were also conducted on the conformations of citric acid and caffeine. As can be seen in Table 3 ▸, the conformation of citric acid is notably more stable in KIGKER com­pared to KIGKER01, which is itself more stable com­pared to CAF–CA. Inter­estingly, caffeine exhibits a com­parable conformation in all three co­crystals.

Thus, the KIGKER cocrystal, given the very energetic nature of the synthesis by milling, is the more stable polymorphic form, followed by the KIGKER01 cocrystal synthesized by slow evaporation. As can be seen in Fig. 8 ▸, KIGKER has four types of hydrogen bonds (O6—H3⋯O5, O1—H1⋯O8, O7—H4⋯O9 and O4—H2⋯N3), resulting in higher stability than KIGKER01, which has three types of hydrogen bonds (O7—H4⋯O5, O4—H2⋯O9 and O6—H3⋯N3). It is therefore not surprising that KIGKER and KIGKER01 are more stable than CAF–CA, with a higher melting tem­per­a­ture, since CAF–CA has only two types of hydrogen bonds (O6—H3⋯O2 and O7—H4⋯N3), as seen in Fig. 6 ▸. A com­parison between the hydrogen bonds of CAF–CA, KIGKER and KIGKER01 is summarized in Table 3 ▸. Finally, the synthesis method used influences the network of hydrogen bonds formed, which itself influences the melting point of the cocrystal. A clear correlation is observed between the density of the hydrogen bonds of the cocrystal and the melting point.

## Supplementary Material

Crystal structure: contains datablock(s) global, I. DOI: 10.1107/S205322962400319X/fp3096sup1.cif

Structure factors: contains datablock(s) I. DOI: 10.1107/S205322962400319X/fp3096Isup2.hkl

Additional figures. DOI: 10.1107/S205322962400319X/fp3096sup3.pdf

Supporting information file. DOI: 10.1107/S205322962400319X/fp3096Isup4.cml

CCDC reference: 2348459

## Figures and Tables

**Table 1:** Crystallographic data, profile and structural parameters for the CAF–CA cocrystal obtained after Rietveld refinement

Crystal data	
Chemical formula	C_14_H_18_N_4_O_9_
*M*_r_	772.6
Cell setting, space group	Triclinic, *P* 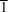
Tem­per­a­ture (K)	293
*a*, *b*, *c* (Å)	14.6803 (3), 8.8743 (2), 6.9537 (7)
α, β, γ (°)	106.9221 (1), 96.304 (1), 97.550 (1)
*V* (Å^3^)	848.64 (2)
*Z*	1
*F*(000)	404
μ (mm^−1^)	0.128
Specimen shape, size (mm)	Cylinder, 0.5
2θ range (°)	2.5–50°
	
Data collection	
Beamline	CRISTAL (SOLEIL)
Specimen mounting	0.5 mm diameter Lindemann capillary
Data collection mode	Transmission
Scan method	Continuous scan
Radiation type	Synchrotron 17 KeV, λ = 0.7289 Å
Binning size (°2θ)	0.004
	
Refinement	
*R* factors and goodness of fit	*R* = 0.069, *Rwp*_nb_ = 0.109, *R*_exp _ = 0.020

**Table 2:** Comparison of the lattice parameters (Å, °) of the CAF–CA cocrystal obtained in this work and CAF–CA co­crystals KIGKER and KIGKER01

Structure	*a*	*b*	*c*	α	β	γ	*V* (Å^3^)	Symmetry	Reference
CAF–CA	14.6803	8.8743	6.9537	106.922	96.304	97.55	848.639	Triclinic *P* 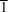	This work
KIGKER	7.38740	8.3967	13.5053	91.333	99.040	99.588	814.72	Triclinic *P* 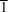	Karki *et al.* (2007 ▸)
KIGKER01	13.7783	12.3149	9.6587	90	92.854	90	1636.84	Monoclinic *P*2_1_/*c*	Smit & Hagen (2015 ▸)

**Table 3:** Comparison of the melting tem­per­a­ture (*T*_m_), hydrogen bonds and ground-state DFT calculations between the CAF–CA cocrystal obtained in this work and CAF–CA co­crystals KIGKER and KIGKER01

	*T*_m_ (°C)	Hydrogen bond			Energy (eV)		
		Number	Type	Distance (Å)	Crystal	Caffeine	Citric acid
CAF–CA	135.0	2	O7—H4⋯N3	1.681	−1910972.06	−1994223.05	−1994223.05
			O6—H3⋯O2	1.650			
KIGKER01	158.9	3	O6—H3⋯N3	1.827	−1911084.85	−1785066.00	−1994338.84
			O4—H2⋯O9	1.690			
			O7—H4⋯O5	1.753			
KIGKER	161.0	4	O4—H2⋯N3	1.848	−1911085.92	−1785064.07	−1994352.35
			O6—H3⋯O5	2.001			
			O7—H4⋯O9	1.862			
			O1—H1⋯O8	2.103			
