# Supplementary material for: Relationship between synthesis method–crystal structure–melting properties in co­crystals: the case of caffeine–citric acid
Source: Acta Crystallogr C Struct Chem. 2024 May 7;80(Pt 6):221–9. doi: 10.1107/S205322962400319X (PMC11150877; doi:10.1107/S205322962400319X)
Supplement: Supplementary file 3 [file c-80-00221-sup3.pdf]

STRUCTURAL CHEMISTRY

Volume 80 (2024)

Supporting information for article:

Relationship between synthesis method–crystal structure–melting properties in cocrystals: the case of caffeine–citric acid

Mathieu Guerain, Hubert Chevreau, Yannick Guinet, Laurent Paccou, Erik Elkaïm and Alain Hédoux

a) GALLOP

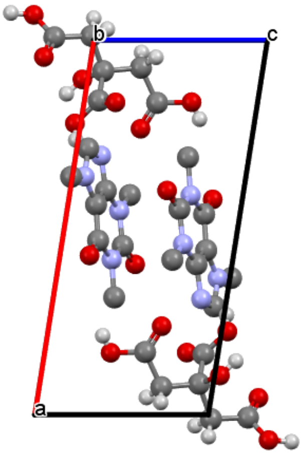

b) FOX

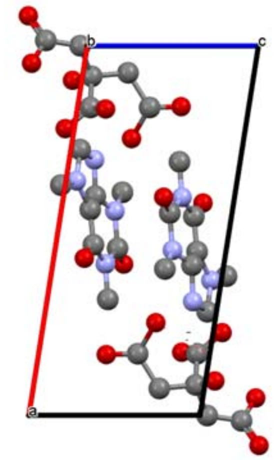

c) DASH

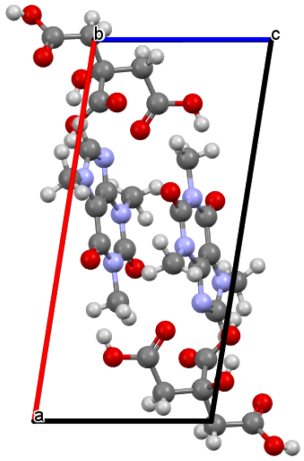

Figure S1 : Best solution for the CAF-CA co-crystal structure obtained by a) GALLOP (local-optimization algorithm), b) FOX (parallel tempering algorithm) and c) DASH (simulated annealing algorithm). View along the *b* axis.

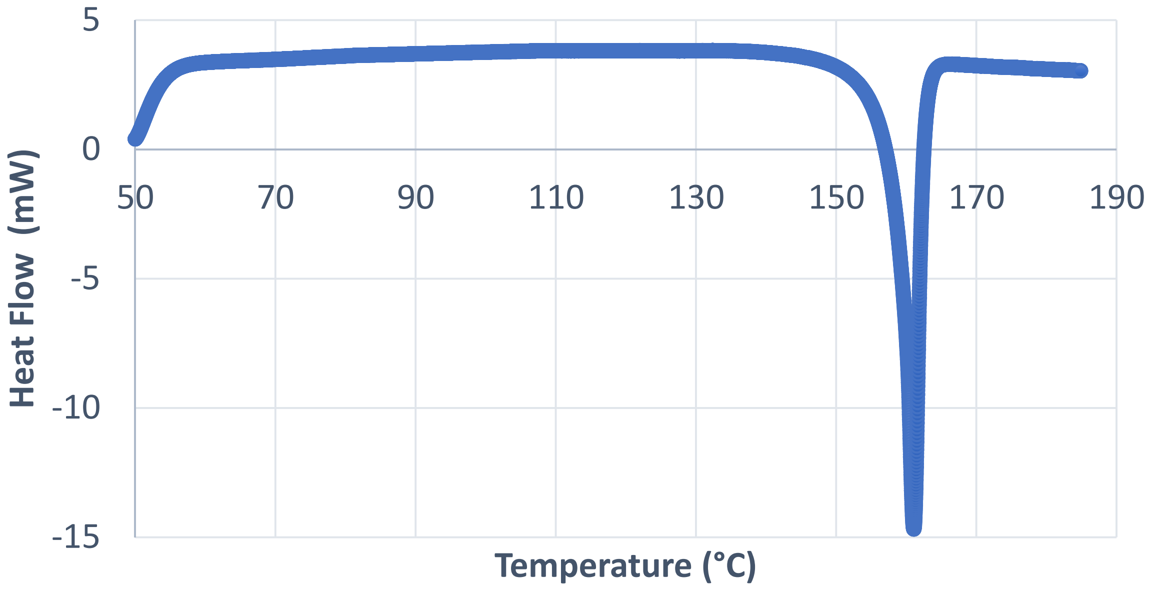

Figure S2 : Calorimetric traces of Caffeine-Citric acid cocrystal obtained by milling
